# Escalated Maximum Dose in the Planning Target Volume Improves Local Control in Stereotactic Body Radiation Therapy for T1-2 Lung Cancer

**DOI:** 10.3390/cancers14040933

**Published:** 2022-02-13

**Authors:** Takaya Inagaki, Hiroshi Doi, Naoko Ishida, Aritoshi Ri, Saori Tatsuno, Yutaro Wada, Takuya Uehara, Masahiro Inada, Kiyoshi Nakamatsu, Makoto Hosono, Yasumasa Nishimura

**Affiliations:** Department of Radiation Oncology, Kindai University Faculty of Medicine, 377-2, Ohno-higashi, Osaka-Sayama 589-8511, Osaka, Japan; inagaki@wakayama-med.ac.jp (T.I.); naoko.ishida@med.kindai.ac.jp (N.I.); 2024e2@med.kindai.ac.jp (A.R.); saorin823st@med.kindai.ac.jp (S.T.); wada.yutarou@med.kindai.ac.jp (Y.W.); ueharatakuya0413@med.kindai.ac.jp (T.U.); im022012@med.kindai.ac.jp (M.I.); kiyoshi@med.kindai.ac.jp (K.N.); hosono@med.kindai.ac.jp (M.H.); ynishi@med.kindai.ac.jp (Y.N.)

**Keywords:** stereotactic body radiation therapy, stereotactic body ablative radiotherapy, lung cancer, dose escalation, squamous cell carcinoma

## Abstract

**Simple Summary:**

Stereotactic body radiotherapy (SBRT) is a treatment option for early-stage lung cancer. The purpose of this study was to investigate the optimal dose distribution and prognostic factors for local control (LC) in 100 patients with lung cancer who underwent SBRT. The 1- and 3-year LC rates were 95.7% and 87.7%, respectively. In summary, we found that squamous cell carcinoma (SQ), T2 tumor stage, and a lower radiotherapy dose were associated with poorer LC in lung cancer. The lower rate of LC in patients with SQ vs. non-SQ was limited to cases with a Dmax below 125 Gy (BED_10_).

**Abstract:**

Stereotactic body radiotherapy (SBRT) is a treatment option for early-stage lung cancer. The purpose of this study was to investigate the optimal dose distribution and prognostic factors for local control (LC) after SBRT for lung cancer. A total of 104 lung tumors from 100 patients who underwent SBRT using various treatment regimens were analyzed. Dose distributions were corrected to the biologically effective dose (BED). Clinical and dosimetric factors were tested for association with LC after SBRT. The median follow-up time was 23.8 months (range, 3.4–109.8 months) after SBRT. The 1- and 3-year LC rates were 95.7% and 87.7%, respectively. In univariate and multivariate analyses, pathologically confirmed squamous cell carcinoma (SQ), T2 tumor stage, and a Dmax < 125 Gy (BED_10_) were associated with worse LC. The LC rate was significantly lower in SQ than in non-SQ among tumors that received a Dmax < 125 Gy (BED_10_) (*p* = 0.016). However, there were no significant differences in LC rate between SQ and non-SQ among tumors receiving a Dmax ≥ 125 Gy (BED_10_) (*p* = 0.198). To conclude, SQ, T2 stage, and a Dmax < 125 Gy (BED_10_) were associated with poorer LC. LC may be improved by a higher Dmax of the planning target volume.

## 1. Introduction

Stereotactic body radiation therapy (SBRT), also known as stereotactic ablative radiation therapy, has been recommended as a therapeutic modality for medically inoperable early-stage non-small-cell lung cancer [1]. The major feature distinguishing SBRT from conventional radiation treatment is the delivery of large radiation doses in a few fractions, resulting in a high biologically effective dose (BED) [2,3]. The use of high-precision techniques is critical for the administration of SBRT, and large dose gradients can be located on the target to achieve maximum therapeutic efficacy while minimizing toxicity to normal tissue [4].

SBRT for extra-cranial tumors was developed by Blomgren et al. in the 1990s [5], and the techniques and clinical evidence of the efficacy of SBRT have dramatically strengthened since then. Suitable fixation methods, respiratory management techniques, and dose calculation algorithms have also been improved to maximize precision and minimize errors. Moreover, both non-coplanar three-dimensional conformal multiple-beam irradiation techniques and intensity-modulated radiotherapy have recently been used to improve the homogeneity of radiation doses and reduce doses to organs at risk [4]. SBRT is now widely accepted as a treatment option for early-stage lung tumors and achieves 80–97% local control (LC) rates by using a BED_10_ of >100 Gy [6,7,8,9,10,11,12].

Despite SBRT being a promising treatment option for malignant solitary pulmonary nodules in clinical practice, the optimal treatment regimen, including the optimal prescribed dose, is poorly understood [8,12,13,14]. In addition, the European Society for Radiation and Oncology-Advisory Committee in Radiation Oncology Practice (ESTRO-ACROP) consensus guidelines recommend that the maximum dose (Dmax) of the planning target volume (PTV) should range from 125% to 150% of the prescription dose [13]. However, the optimal dose distribution and clinical evidence for the benefit of elevated maximum doses for tumors are unclear. The purpose of this study was to investigate the optimal dose distribution and prognostic factors for LC after SBRT for lung cancer by studying patients who received SBRT through various treatment regimens at a single institute.

## 2. Materials and Methods

Clinical diagnosis and indications for SBRT were decided based on clinical information, images, and pathological diagnosis from the Thoracic Tumor Board Conference of the Kindai University Hospital (Osaka, Japan). The panel consisted of pulmonologists, medical oncologists, thoracic surgeons, radiation oncologists, and radiologists.

A total of 129 patients (136 tumors) who received SBRT for lung cancer at Kindai University Hospital between January 2008 and May 2021 were included in this retrospective study. SBRT was defined as definitive radiotherapy for primary tumors in fewer than 10 fractions. We confirmed the clinical stage at the initial diagnosis based on the 8th edition of the International Union Against Cancer/American Joint Committee on Cancer tumor-node-metastasis classification system [15]. Patients whose tumors were in contact with the pleura and did not show respiratory migration on four-dimensional computed tomography (CT) were diagnosed with stage T3 cancer (chest wall invasion). We excluded the following patients: 15 patients with stage T3 cancer (*n* = 13, chest wall invasion; *n* = 2, another tumor in the same lobe), 5 (8 tumors) with metastatic lung tumors, 1 with small-cell lung cancer, and 8 who had a follow-up duration of less than 6 months without any specific events. Therefore, 100 patients (104 tumors) who underwent SBRT were analyzed. Patient characteristics are summarized in Table 1. One patient with Eastern Cooperative Oncology Group Performance Status 4 due to amyotrophic lateral sclerosis who received SBRT after careful consideration was included in this study because he was expected to have a long-term prognostic benefit from SBRT. The thoracic surgeon diagnosed 19 tumors as operable, but SBRT was performed at the patients’ request. Thirty-five tumors were pathologically diagnosed as adenocarcinoma and twenty-two as squamous cell carcinoma (SQ). Five tumors were pathologically diagnosed as malignant, but the histological type could not be diagnosed. Forty-two tumors could not be pathologically diagnosed but were diagnosed as carcinoma from two or more continuous growths detected by CT or ^18^F-fluorodeoxyglucose (^18^FDG) uptake on ^18^FDG-positron emission tomography (PET)/CT.

### 2.1. Radiotherapy

All patients were helically scanned in the supine position using an Aquilion Prime (CANON Medical Systems, Tochigi, Japan) CT unit between January 2008 and October 2015 and an Optima CT660 (GE Healthcare, Chicago, IL, USA) CT unit from October 2015 onwards. The CT data were transferred to the treatment planning system to outline the volumes of interest. The clinical target volume (CTV) was created from the gross tumor volume (GTV) by adding 5–8 mm margins between January 2008 and March 2018, and the CTV was equal to the GTV from March 2018 onwards. Four-dimensional CT (4D-CT) was performed in order to evaluate the respiratory migration of each tumor in all patients. As necessary, the internal target volume (ITV) was created from the CTV by adding a sufficient margin on 4D-CT. The PTV was calculated by adding a margin of 5 mm to each CTV or ITV. All treatment plans were created using the Eclipse treatment planning system (Varian Medical Systems, Palo Alto, CA, USA). SBRT was performed using three-dimensional conformal radiation therapy (3D-CRT) or volumetric modulated arc therapy (VMAT), which typically used 6 MV X-rays. The planned radiotherapy was delivered using Clinac 21EX (Varian Medical Systems, Palo Alto, CA, USA) between January 2008 and October 2015 and a TrueBeam linear accelerator (Varian Medical Systems, Palo Alto, CA, USA) from October 2015 onwards. SBRT was performed using a VMAT technique following the replacement of the linear accelerator.

The prescribed dose was calculated for delivery to a reference point in 3D-CRT and was normalized to 95% of the PTV in VMAT. The dose distributions were calculated using the pencil beam convolution (PBC, Eclipse), analytical anisotropic (AAA, Eclipse), or Acuros XB (Eclipse) algorithms. Thirty-seven tumors were treated with 3D-CRT with a median of 48 Gy (range, 48–52 Gy) in a median of four fractions (range, four to five fractions) to the isocenter. Twelve tumors were treated with 3D-CRT with a median of 45 Gy (range, 44.4–47.5 Gy) in four fractions prescribed to the 86% isodose line covering the PTV. One tumor was treated with 3D-CRT with a prescribed dose of 42 Gy in four fractions to cover 95% of the PTV (D95). Finally, 54 tumors were treated with VMAT with a prescribed median dose (D95) of 48 Gy (range, 42–60 Gy) in a median of four fractions (range, four to eight fractions). Table 2 summarizes the prescribed methods. Six tumors were within 2 cm of the mediastinum. Of these, one and five tumors were treated with 50 Gy in five fractions using the 3D-CRT technique and 60 Gy in eight fractions using the VMAT technique, respectively. The remaining 98 tumors were treated in four fractions.

All treatment plans calculated using the PBC or Acuros XB algorithms were recalculated using the AAA algorithm. To compare the various prescribed methods, we reviewed the dose-volume histogram (DVH) parameters of all clinical plans. Dmax, dose to the 50% volume (D50), D95, D98, and minimum dose (Dmin) were confirmed from the DVH parameters for the PTV. Considering the different number of fractions, we thought it would be reasonable to calculate and compare each DVH parameter to the BED using the linear-quadratic formula with an assumed α/β ratio of 10 Gy for tumors. Each dose was divided by the number of fractions to calculate the dose of one fraction. Using the number of fractions and the dose of one fraction, we calculated the BED_10_. The calculation Formula (1) for BED is as follows:

BED = n × d × [1 + d/(α/β)](1)

where n is the number of fractions, d is the dose of one fraction, and the value of α/β is 10 Gy for the tumors [12]. Table 3 summarizes the DVH parameters in terms of BED_10_ and the PTV.

### 2.2. Follow-Up

We defined LC as the absence of local failure (LF) in the PTV. LC, survival times, and the time to toxicity were defined as the intervals from the start of SBRT to the date of diagnosis of LF, the date of death, and the date of the occurrence of events due to radiotherapy, respectively. LFs were identified by experienced physicians based on ^18^FDG uptake on ^18^FDG-PET/CT and continuous growth on CT images. Referring to the clinical chart, toxicity was assessed using the Common Terminology Criteria for Adverse Events (CTCAE), version 5.0 [16], and we extracted adverse events of Grade 3 or higher.

### 2.3. Statistical Analysis

The data are expressed as medians with the range in parentheses unless otherwise indicated. The time to a specific event was defined as the interval from the start of radiotherapy to the date of the event. Overall survival (OS) was measured from the start of SBRT until death from any cause (censored at the date of last confirmed survival for surviving patients). Progression-free survival (PFS) was measured from the start of SBRT until the first event of disease progression or death, whichever occurred first (censored at the date of last confirmed survival for patients with no events). LC was measured from the start of SBRT until recurrence within the PTV. Cumulative time was calculated using the Kaplan–Meier method, and the differences in probability curves were assessed using the log-rank test. The Cox proportional-hazards model was used to evaluate factors that influenced LC. The cut-off values of potential predictive factors were decided based on receiver operating characteristic curves. The results were reported as hazard ratios with corresponding 95% confidence intervals (CI). Variables with *p*-values < 0.05 according to univariate analysis were analyzed in the multivariate model using Cox regression analysis. All statistical analyses were performed using GraphPad Prism version 8.4.3 (GraphPad Software, Inc.; San Diego, CA, USA) and the JMP software version 12.2.0 (SAS Institute, Cary, NC, USA). A *p*-value < 0.05 was considered statistically significant.

## 3. Results

### 3.1. Clinical Outcomes after SBRT

**Figure 1:**
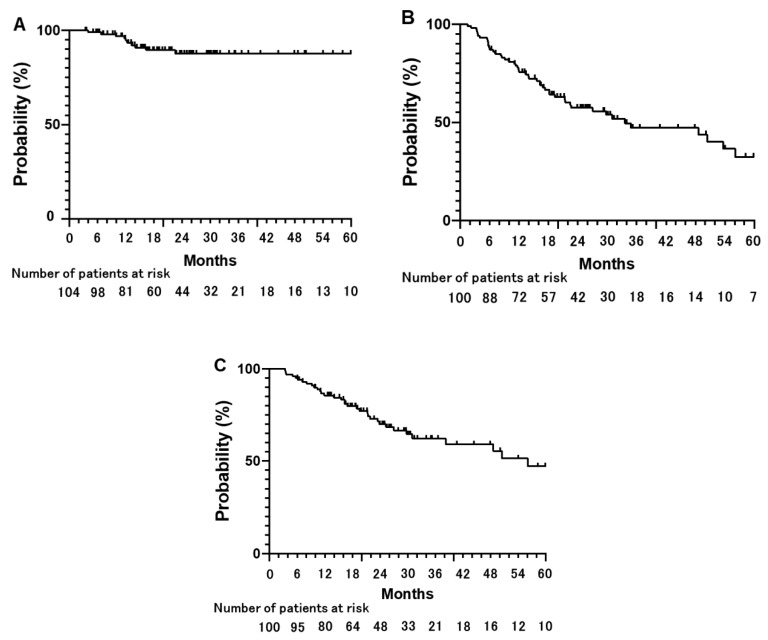
Clinical outcomes after stereotactic body radiotherapy in all eligible tumors and patients. Cumulative rate of local control (LC), progression free survival (PFS), and overall survival (OS) are shown. (**A**) The 1-, 3-, and 5-year LC rates are 95.7%, 87.7%, and 87.7%, respectively. (**B**) The 1-, 3-, and 5-year PFS rates are 75.6%, 47.2%, and 32.4%, respectively. (**C**) The 1-, 3-, and 5-year OS rates are 85.6%, 62.2%, and 47.2%, respectively.

The median follow-up time was 23.8 months (range, 3.4–109.8 months) after SBRT. Ten patients experienced LF at a median follow-up of 12.1 months (range, 4.0–22.4 months). Eight LFs were diagnosed by ^18^FDG uptake on ^18^FDG-PET/CT and continuous growth on CT images, and two LFs were diagnosed by continuous growth on CT images. The 3-year LC rate was 87.7% (95% CI: 78.3–93.3%) for all tumors (Figure 1A). The median PFS and OS times were 33.8 and 56.1 months, respectively (Figure 1B,C). The 3- and 5-year PFS rates were 47.2% (95% CI: 35.7–59.0%) and 32.4% (95% CI: 19.8–48.3%), respectively (Figure 1B). The 3- and 5-year OS rates were 62.2% (95% CI: 50.4–72.8%) and 47.2% (95% CI: 32.3–62.6%), respectively (Figure 1C). Six patients experienced pneumonitis ≥ Grade 3 (5.7%), including two at Grade 5 (1.9%). One patient with Grade 5 pneumonitis had pathologically proven interstitial pneumonia before SBRT. No other Grade ≥ 3 toxicities were observed.

### 3.2. Prognostic Factors for Local Control after SBRT

**Figure 2:**
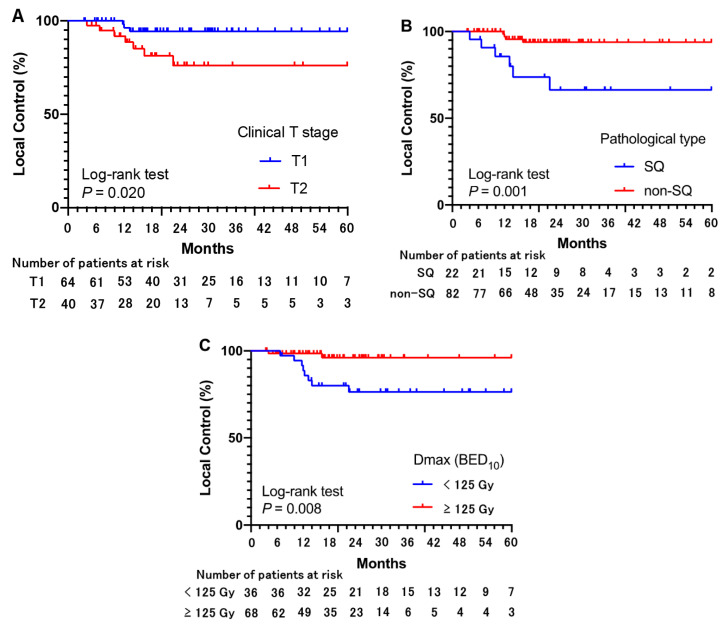
Local control for each factor. Cumulative rates of local control for each risk factor identified in univariate and multivariate analyses using Cox regression are indicated. Poor local control is significantly associated with (**A**) clinical T2 stage, (**B**) pathologically confirmed squamous cell carcinoma (SQ), and (**C**) maximum biologically effective doses (BED_10_) of <125 Gy.

In univariate and multivariate analyses, pathologically confirmed SQ, T2 tumor stage, and a Dmax for the PTV (PTVmax) < 125 Gy (BED_10_) were associated with a worse LC rate (Table 4, Figure 2). In addition, there were no significant differences between pathologically proven adenocarcinoma and pathologically unknown, clinically diagnosed lung cancer (Supplementary Figure S1). 

**Figure 3:**
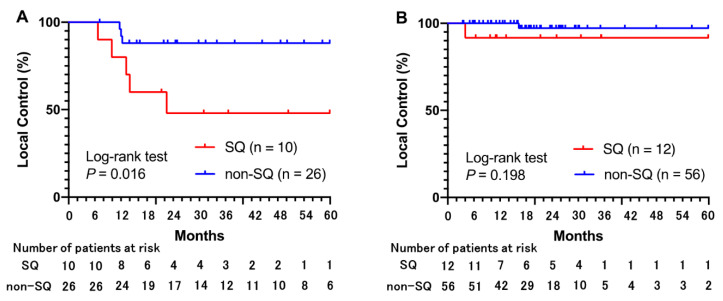

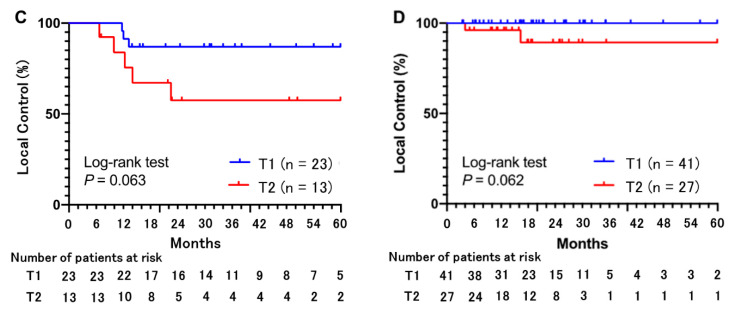
Local control associated with biologically effective doses of <125 Gy and ≥125 Gy. (**A**) For tumors that received a PTVmax < 125 Gy (BED_10_), the 3-year local control (LC) rates are 48.0% for SQ and 88.0% for non-SQ. (**B**) For tumors that received a PTVmax ≥125 Gy (BED_10_), the 3-year LC rates are 91.7% for SQ and 97.2% for non-SQ. (**C**) For tumors that received a PTVmax < 125 Gy (BED_10_), the 3-year LC rates are 87.0% for clinical T1 tumors and 57.5% for clinical T2 tumors. (**D**) For tumors that received a PTVmax ≥ 125 Gy (BED_10_), the 3-year LC rates are 100% for clinical T1 tumors and 89.2% for clinical T2 tumors.

Among 36 tumors that received a PTVmax < 125 Gy (BED_10_), the LC rate was significantly lower for SQ than for non-SQ (Figure 3A). Among 68 tumors that received a PTVmax ≥ 125 Gy (BED_10_), there were no significant differences in the LC rate between SQ and non-SQ (Figure 3B). The differences in LC between tumors of stage T1 or T2 were smaller (but not significant) in the PTVmax ≥125 Gy (BED_10_) group than in the PTVmax < 125 Gy (BED_10_) group (Figure 3C,D).

## 4. Discussion

In the present study, pathologically confirmed SQ, T2 tumor stage, and a PTVmax < 125 Gy (BED_10_) were associated with a worse LC rate in patients with stage T1 or T2 lung cancer who received SBRT. A PTVmax > 125 Gy (BED_10_) was significantly associated with better LC; Dmin, D98%, D95%, and D50% of the PTV were not associated with LC. The ESTRO-ACROP consensus guidelines state that the Dmax of the PTV should range from 125% to 150% of the prescription dose [13]. However, to our knowledge, there are only a few reports describing that an escalated Dmax in the PTV is associated with a better LC. Onishi et al. reported that better LC was associated with a prescribed BED_10_ > 100 Gy [7]. Further, Koshy et al. reported that better OS was associated with a prescribed BED_10_ > 150 Gy [17]. In these previous reports, SBRT had conventional isocentric prescriptions rather than modern volume-based prescriptions. Kestin et al. reported that a Dmean > 125 Gy for the PTV was associated with better LC [18].

Pathologically confirmed SQ was associated with poor LC in our study. The role of histopathological subtype in this context has only been reported recently. Previous reports showed that SQ was associated with LF in lung tumors receiving SBRT [19,20,21]. Using a large database, Parzen et al. reported that SBRT regimens with a BED_10_ > 150 Gy may confer a survival benefit in patients with SQ [22]. However, the recommended dose distribution in SBRT for lung SQ is still unclear. In our study, a PTVmax ≥ 125 Gy seemed to improve LC of SQ, and a dose–response relationship was assumed to exist for lung SQ. However, the molecular mechanism of radioresistance in SQ is less clear. Ren et al. reported that hypoxia-inducible factor-1α (HIF-1α) was expressed more in SQ than in adenocarcinoma. Positivity for HIF-1α in the tumor tissues of patients corresponded to lower OS [23]. Hypoxia may have led to a decrease in the LC rate of SQ [3]. Further clinical studies, including molecular analyses, are warranted to clarify the mechanism of the poorer LC of SQ after SBRT and for a potential optimal combined therapy.

SBRT is a viable treatment option for both stage T1 and T2 primary lung cancer [1]. However, a worse LC rate has been reported for SBRT for stage T2 tumors [8,12], which are tumors measuring between 3 and 5 cm or tumors involving the main bronchus without the carina or invasion of the visceral pleura or that are associated with atelectasis or obstructive pneumonitis [15]. In addition, stage T2 tumors have been excluded from several prospective clinical trials focused on outcomes after SBRT [11,24]. Tumor size is potentially associated with hypoxia, which leads to radioresistance [25,26]. Patients with stage T2 tumors who received a PTVmax ≥ 125 Gy (BED_10_) showed a trend toward improved LC, yet there were no significant differences in comparison with those who received a PTVmax < 125 Gy (BED_10_). In this study, tumors were classified as stage T2 due to an invasion of the visceral pleurae for 26 tumors (65%) and having a diameter ≥ 3 cm for 14 tumors (35%). The complexity of stage T2 lung cancer may undermine the efficacy of a high PTVmax. Further investigation using more intensive treatment strategies such as dose escalation and combination with chemotherapy may be needed to improve LC of stage T2 tumors [8,12].

The CTV margin was not significantly associated with LC, PFS, or OS in this study. The American Society for Therapeutic Radiology and Oncology, the American College of Radiology Practice Guidelines, and the ESTRO-ACROP consensus guidelines recommend that no CTV margin be added to GTV for SBRT [13,27]. In studies by the Japan Clinical Oncology Group (JCOG), GTV is commonly equated with CTV [6,24,28]. However, microscopic tumor extension can lead to LF after SBRT with a minimized margin for the GTV [29]. In addition, Giraud et al. reported that a CTV margin of 8 mm for adenocarcinoma and 6 mm for SQ would cover 95% of microscopic tumor spread based on the pathologic evaluation of surgically resected specimens [30]. In the present study, the CTV margin added to the GTV did not improve LC. Because the addition of a CTV margin was deemed unnecessary, microscopic tumors might be treated with moderate doses to the area surrounding the PTV for photon SBRT.

We acknowledge that there are several limitations to this study. We presented oncologic outcomes after SBRT for primary lung cancer along with predictive factors in a limited number of patients with a relatively short follow-up period. However, the median follow-up was approximately 24 months after SBRT, which can be sufficient to evaluate the outcomes. In addition, we included 100 patients in this study and presented the predictive factors in a multivariate analysis. In this study, pathologically proven SQ, pathologically proven adenocarcinoma, and pathologically unknown, clinically diagnosed lung cancer were diagnosed in 22 (21.2%), 35 (33.7%), and 47 (45.2%) tumors, respectively. However, there were no significant differences between pathologically confirmed adenocarcinoma and pathologically unknown, clinically diagnosed lung cancer (Supplementary Materials). In addition, poorer LC of SQ after SBRT seems to be consistent with previous reports [19,20,21]. Further, there are no apparent data from a prospective clinical trial in which the benefit of dose-escalated Dmax for the PTV contributed to a better LC. The JCOG 1408 trial, which is ongoing, is a randomized Phase III trial assessing SBRT for stage IA primary lung cancer that is comparing an escalated dose of 55 Gy in four fractions against standard doses of 42 Gy in four fractions [24]. We hope that the JCOG 1408 trial will demonstrate an improvement in LC with dose escalation.

## 5. Conclusions

In conclusion, SQ and T2 tumor stages were independently associated with poor LC after SBRT for patients with lung cancer. Furthermore, the escalated PTVmax to BED_10_ > 125 Gy can improve LC with SBRT for lung cancer.

## Figures and Tables

**Table 1:** Patients’ characteristics.

Factors	*n* = 100	(%)
Age (years), median (range)	77.5 (50–91)	
Sex		
Male	67	(67.0)
Female	33	(33.0)
ECOG-PS		
0	53	(53.0)
1	27	(27.0)
2	16	(16.0)
3	3	(3.0)
4	1	(1.0)
Tobacco-smoking history		
Never	22	(22.0)
Former	71	(71.0)
Current	7	(7.0)
Smoking (pack-years), median (range)	54 (0.5–136)	
Simultaneous primary cancer	9	(9.0)
Interstitial pneumonia	10	(10.0)
Thoracic surgery history	31	(31.0)
**Tumors**	***n* = 104**	**(%)**
Operability		
Operable	19	(18.3)
Inoperable	85	(81.7)
Pathological diagnosis		
Adenocarcinoma	35	(33.7)
Squamous cell carcinoma	22	(21.2)
Unknown	47	(45.2)
Tumor location		
Left upper lobe	31	(29.8)
Left lower lobe	8	(7.7)
Right upper lobe	26	(25.0)
Right middle lobe	7	(6.7)
Right lower lobe	32	(30.8)
Clinical T stage		
Tmi	1	(1.0)
T1a	8	(7.7)
T1b	40	(38.5)
T1c	15	(14.4)
T2a	10	(9.6)
T2b	4	(3.8)
T2 (visceral pleural invasion)	26	(25.0)

Abbreviations: ECOG-PS, Eastern Cooperative Oncology Group Performance Status.

**Table 2:** Summary of the prescription methods.

Technique	Median Prescription Dose (Range)	Median Fractions (Range)	Prescription	Number of Tumors	(%)
3D-CRT	48 Gy (48.0–52.0)	4 (4–5)	Isocenter	37	(35.6)
3D-CRT	45 Gy (44.4–47.5)	4 (4)	Covering PTV of 86% isodose line	12	(11.5)
3D-CRT	42 Gy (42.0)	4 (4)	PTV D95	1	(1.0)
VMAT	48 Gy (42.0–60.0)	4 (4–8)	PTV D95	54	(51.9)

Abbreviations: 3D-CRT, three-dimensional conformal radiation therapy; VMAT, volumetric modulated arc therapy; D95, dose to the 95-percentage volume of the region of interest; PTV, planning target volume.

**Table 3:** Summary of dose histogram parameters.

PTV	Median (Range)
Volume (cm^3^)	36.8 (7.2–190.7)
Dmax (BED_10_, Gy)	134.9 (106.0–191.2)
D50 (BED_10_, Gy)	117.1 (96.5–146.0)
D95 (BED_10_, Gy)	105.4 (86.1–130.7)
D98 (BED_10_, Gy)	101.5 (82.4–126.2)
Dmin (BED_10_, Gy)	93.8 (52.7–109.6)

Abbreviations: Dmax, maximum dose; BED, biologically effective dose; Dx, dose to the x-percentage volume of the region of interest; Dmin, minimum dose; PTV, planning target volume.

**Table 4:** Univariate and multivariate analyses for the factors associated with local control.

			Univariate Analysis		Multivariate Analysis	
Factors	*n* (%)	Number of Events	Hazard Ratio (95% CI)	*p*-Value	Hazard Ratio (95% CI)	*p*-Value
Age (y)						
<80	68 (65.4%)	7	1	0.855		
≥80	36 (34.6%)	3	1.135 (0.228–3.414)			
Sex						
Male	71 (68.3%)	9	1	0.12		
Female	33 (31.7%)	1	0.194 (0.652–40.721)			
ECOG-PS						
0, 1	83 (79.8%)	7	1	0.342		
2–4	21 (20.2%)	3	1.929 (0.497–7.487)			
Smoking history						
None	22 (21.2%)	3	1	0.586		
Any	82 (78.8%)	7	1.456 (0.377–5.633)			
Operability						
Operable	19 (18.3%)	1	1	0.508		
Inoperable	85 (81.7%)	9	2.010 (0.255–15.864)			
Pathological diagnosis						
Squamous cell carcinoma	22 (21.2%)	6	1	0.005	1	0.025
other	82 (78.8%)	4	0.160 (0.045–0.567)		0.228 (0.063–0.828)	
Clinical T Stage						
T1	64 (61.5%)	3	1	0.033	1	0.042
T2	40 (38.5%)	7	4.369 (1.128–16.915)		4.111 (1.054–16.042)	
CTV margin						
0 mm (GTV = CTV)	41 (39.4%)	2	1	0.219		
≥1 mm	63 (60.6%)	8	2.648 (0.560–12.527)			
PTV volume						
<40 cm^3^	57 (54.8%)	3	1	0.068		
≥40 cm^3^	47 (45.2%)	7	3.536 (0.912–13.704)			
Dmax (BED_10_, Gy)						
<125 Gy	36 (34.6%)	8	1	0.021	1	0.041
≥125 Gy	68 (65.4%)	2	0.161(0.034–0.758)		0.195 (0.040–0.945)	
D50 (BED_10_, Gy)						
<111 Gy	37 (35.6%)	7	1	0.053		
≥111 Gy	67 (64.4%)	3	0.263 (0.068–1.017)			
D95 (BED_10_, Gy)						
<104 Gy	42 (40.4%)	7	1	0.121		
≥104 Gy	62 (59.6%)	3	0.343 (0.089–1.328)			
D98 (BED_10_, Gy)						
<100 Gy	35 (33.7%)	5	1	0.473		
≥100 Gy	69 (66.3%)	5	0.635 (0.183–2.195)			
Dmin (BED_10_, Gy)						
<95 Gy	72 (69.2%)	5	1	0.136		
≥95 Gy	32 (30.8%)	5	2.579 (0.112–1.346)			

Abbreviations: CI, confidence interval; ECOG-PS, Eastern Cooperative Oncology Group Performance Status; Dmax, maximum dose; BED, biologically effective dose; Dx, dose to the x-percentage volume of the region of interest; Dmin, minimum dose; PTV, planning target volume; CTV, Clinical target volume; GTV, gross tumor volume.

## Data Availability

The data are available from the corresponding authors upon reasonable request.

## Supplementary Materials

The following supporting information can be downloaded at: https://www.mdpi.com/article/10.3390/cancers14040933/s1. Figure S1: Cumulative rate of local control per pathological diagnosis. There were significant differences between pathologically proven squamous cell carcinoma (SQ) and pathologically proven adenocarcinoma and pathologically unknown, clinically diagnosed lung cancer (*p* = 0.022 and 0.09, respectively). However, there were no significant differences between pathologically proven adenocarcinoma and pathologically unknown, clinically diagnosed lung cancer (*p* = 0.805).

## References

[1] Ettinger D.S., Wood D.E., Aisner D.L., Akerley W., Bauman J.R., Bharat A., Bruno D.S., Chang J.Y., Chirieac L.R., D’Amico T.A. National Comprehensive Cancer Network. NCCN Clinical Practice Guidelines in Oncology (NCCN Guidelines®®): Non-Small Cell Lung Cancer, Version 1.2022. https://www.nccn.org/professionals/physician_gls/pdf/nscl.pdf.

[2] Nagata Y., Kimura T. (2018). Stereotactic Body Radiotherapy (SBRT) for Stage I Lung Cancer. Jpn. J. Clin. Oncol..

[3] Brown J.M., Carlson D.J., Brenner D.J. (2014). The Tumor Radiobiology of SRS and SBRT: Are More than the 5 Rs Involved?. Int. J. Radiat. Oncol. Biol. Phys..

[4] Sapkaroski D., Osborne C., Knight K.A. (2015). A Review of Stereotactic Body Radiotherapy—Is Volumetric Modulated Arc Therapy the Answer?. J. Med. Radiat. Sci..

[5] Blomgren H., Lax I., Näslund I., Svanström R. (1995). Stereotactic High Dose Fraction Radiation Therapy of Extracranial Tumors Using an Accelerator. Clinical Experience of the First Thirty-One Patients. Acta Oncol..

[6] Nagata Y., Hiraoka M., Shibata T., Onishi H., Kokubo M., Karasawa K., Shioyama Y., Onimaru R., Kozuka T., Kunieda E. (2015). Prospective Trial of Stereotactic Body Radiation Therapy for Both Operable and Inoperable T1N0M0 Non-Small Cell Lung Cancer: Japan Clinical Oncology Group Study JCOG0403. Int. J. Radiat. Oncol. Biol. Phys..

[7] Onishi H., Araki T., Shirato H., Nagata Y., Hiraoka M., Gomi K., Yamashita T., Niibe Y., Karasawa K., Hayakawa K. (2004). Stereotactic Hypofractionated High-Dose Irradiation for Stage I Nonsmall Cell Lung Carcinoma: Clinical Outcomes in 245 Subjects in a Japanese Multiinstitutional Study. Cancer.

[8] Shibamoto Y., Hashizume C., Baba F., Ayakawa S., Miyakawa A., Murai T., Takaoka T., Hattori Y., Asai R. (2015). Stereotactic Body Radiotherapy Using a Radiobiology-Based Regimen for Stage I Non-Small-Cell Lung Cancer: Five-Year Mature Results. J. Thorac. Oncol..

[9] Grills I.S., Hope A.J., Guckenberger M., Kestin L.L., Werner-Wasik M., Yan D., Sonke J.J., Bissonnette J.P., Wilbert J., Xiao Y. (2012). A Collaborative Analysis of Stereotactic Lung Radiotherapy Outcomes for Early-Stage Non-Small-Cell Lung Cancer Using Daily Online Cone-Beam Computed Tomography Image-Guided Radiotherapy. J. Thorac. Oncol..

[10] Chang J.Y., Senan S., Paul M.A., Mehran R.J., Louie A.V., Balter P., Groen H.J.M., McRae S.E., Widder J., Feng L. (2015). Stereotactic Ablative Radiotherapy Versus Lobectomy for Operable Stage I Non-Small-Cell Lung Cancer: A Pooled Analysis of Two Randomised Trials. Lancet Oncol..

[11] Chang J.Y., Mehran R.J., Feng L., Verma V., Liao Z., Welsh J.W., Lin S.H., O’Reilly M.S., Jeter M.D., Balter P.A. (2021). Stereotactic Ablative Radiotherapy for Operable Stage I Non-Small-Cell Lung Cancer (Revised STARS): Long-Term Results of a Single-Arm, Prospective Trial with Prespecified Comparison to Surgery. Lancet Oncol..

[12] Lee P., Loo B.W., Biswas T., Ding G.X., El Naqa I.M., Jackson A., Kong F.M., LaCouture T., Miften M., Solberg T. (2021). Local Control After Stereotactic Body Radiation Therapy for Stage I Non-Small Cell Lung Cancer. Int. J. Radiat. Oncol. Biol. Phys..

[13] Guckenberger M., Andratschke N., Dieckmann K., Hoogeman M.S., Hoyer M., Hurkmans C., Tanadini-Lang S., Lartigau E., Méndez Romero A., Senan S. (2017). ESTRO ACROP Consensus Guideline on Implementation and Practice of Stereotactic Body Radiotherapy for Peripherally Located Early Stage Non-Small Cell Lung Cancer. Radiother. Oncol..

[14] Shibamoto Y., Otsuka S., Iwata H., Sugie C., Ogino H., Tomita N. (2012). Radiobiological Evaluation of the Radiation Dose as Used in High-Precision Radiotherapy: Effect of Prolonged Delivery Time and Applicability of the Linear-Quadratic Model. J. Radiat. Res..

[15] Brierley J.D., Gospodarowicz M.K., Wittekind C.H. (2017). TNM Classification of Malignant Tumours.

[16] US Department of Health and Human Services National Institutes of Health—National Cancer Institute Common Terminology Criteria for Adverse Events (CTCAE) Version 5.0. https://ctep.cancer.gov/protocoldevelopment/electronic_applications/docs/ctcae_v5_quick_reference_5x7.pdf.

[17] Koshy M., Malik R., Weichselbaum R.R., Sher D.J. (2015). Increasing Radiation Therapy Dose Is Associated with Improved Survival in Patients Undergoing Stereotactic Body Radiation Therapy for Stage I Non-Small-Cell Lung Cancer. Int. J. Radiat. Oncol. Biol. Phys..

[18] Kestin L., Grills I., Guckenberger M., Belderbos J., Hope A.J., Werner-Wasik M., Sonke J.J., Bissonnette J.P., Xiao Y., Yan D. (2014). Dose–Response Relationship with Clinical Outcome for Lung Stereotactic Body Radiotherapy (SBRT) Delivered via Online Image Guidance. Radiother. Oncol..

[19] Shiue K., Cerra-Franco A., Shapiro R., Estabrook N., Mannina E.M., Deig C.R., Althouse S., Liu S., Wan J., Zang Y. (2018). Histology, Tumor Volume, and Radiation Dose Predict Outcomes in NSCLC Patients After Stereotactic Ablative Radiotherapy. J. Thorac. Oncol..

[20] Woody N.M., Stephans K.L., Andrews M., Zhuang T., Gopal P., Xia P., Farver C.F., Raymond D.P., Peacock C.D., Cicenia J. (2017). A Histologic Basis for the Efficacy of SBRT to the Lung. J. Thorac. Oncol..

[21] Hörner-Rieber J., Bernhardt D., Dern J., König L., Adeberg S., Paul A., Heussel C.P., Kappes J., Hoffmann H., Herth F.J.P. (2017). Histology of Non-Small Cell Lung Cancer Predicts the Response to Stereotactic Body Radiotherapy. Radiother. Oncol..

[22] Parzen J.S., Almahariq M.F., Quinn T.J., Siddiqui Z.A., Thompson A.B., Guerrero T., Lee K., Stevens C., Grills I.S. (2021). Higher Biologically Effective Dose Is Associated with Improved Survival in Patients with Squamous Cell Carcinoma of the Lung Treated with Stereotactic Body Radiation Therapy. Radiother. Oncol..

[23] Ren W., Mi D., Yang K., Cao N., Tian J., Li Z., Ma B. (2013). The Expression of Hypoxia-Inducible Factor-1α and Its Clinical Significance in Lung Cancer: A Systematic Review and Meta-Analysis. Swiss Med. Wkly..

[24] Kimura T., Nagata Y., Eba J., Ozawa S., Ishikura S., Shibata T., Ito Y., Hiraoka M., Nishimura Y., Radiation Oncology Study Group of the Japan Clinical Oncology Group (2017). A Randomized Phase III Trial of Comparing Two Dose-Fractionations Stereotactic Body Radiotherapy (SBRT) for Medically Inoperable Stage IA Non-Small Cell Lung Cancer or Small Lung Lesions Clinically Diagnosed as Primary Lung Cancer: Japan Clinical Oncology Group Study JCOG1408 (J-SBRT Trial). Jpn. J. Clin. Oncol..

[25] Dunst J., Stadler P., Becker A., Lautenschläger C., Pelz T., Hänsgen G., Molls M., Kuhnt T. (2003). Tumor Volume and Tumor Hypoxia in Head and Neck Cancers. The Amount of the Hypoxic Volume Is Important. Strahlenther. Onkol..

[26] Petrova V., Annicchiarico-Petruzzelli M., Melino G., Amelio I. (2018). The Hypoxic Tumour Microenvironment. Oncogenesis.

[27] Potters L., Kavanagh B., Galvin J.M., Hevezi J.M., Janjan N.A., Larson D.A., Mehta M.P., Ryu S., Steinberg M., Timmerman R. (2010). American Society for Therapeutic Radiology and Oncology (ASTRO) and American College of Radiology (ACR) Practice Guideline for the Performance of Stereotactic Body Radiation Therapy. Int. J. Radiat. Oncol. Biol. Phys..

[28] Onimaru R., Onishi H., Ogawa G., Hiraoka M., Ishikura S., Karasawa K., Matsuo Y., Kokubo M., Shioyama Y., Matsushita H. (2018). Final Report of Survival and Late Toxicities in the Phase I Study of Stereotactic Body Radiation Therapy for Peripheral T2N0M0 Non-Small Cell Lung Cancer (JCOG0702). Jpn. J. Clin. Oncol..

[29] Uemoto K., Doi H., Shiomi H., Yamada K., Tatsumi D., Yasumoto T., Takashina M., Koizumi M., Oh R.J. (2018). Clinical Assessment of Micro-Residual Tumors During Stereotactic Body Radiation Therapy for Hepatocellular Carcinoma. Anticancer Res..

[30] Giraud P., Antoine M., Larrouy A., Milleron B., Callard P., De Rycke Y., Carette M.F., Rosenwald J.C., Cosset J.M., Housset M. (2000). Evaluation of Microscopic Tumor Extension in Non-Small-Cell Lung Cancer for Three-Dimensional Conformal Radiotherapy Planning. Int. J. Radiat. Oncol. Biol. Phys..

