# Supplementary material for: Escalated Maximum Dose in the Planning Target Volume Improves Local Control in Stereotactic Body Radiation Therapy for T1-2 Lung Cancer
Source: Cancers (Basel). 2022 Feb 13;14(4):933. doi: 10.3390/cancers14040933 (PMC8870557; doi:10.3390/cancers14040933)
Supplement: Supplementary file 1 [file cancers-14-00933-s001.zip › cancers-1573846-supplementary.pdf]

Supplementary Materials

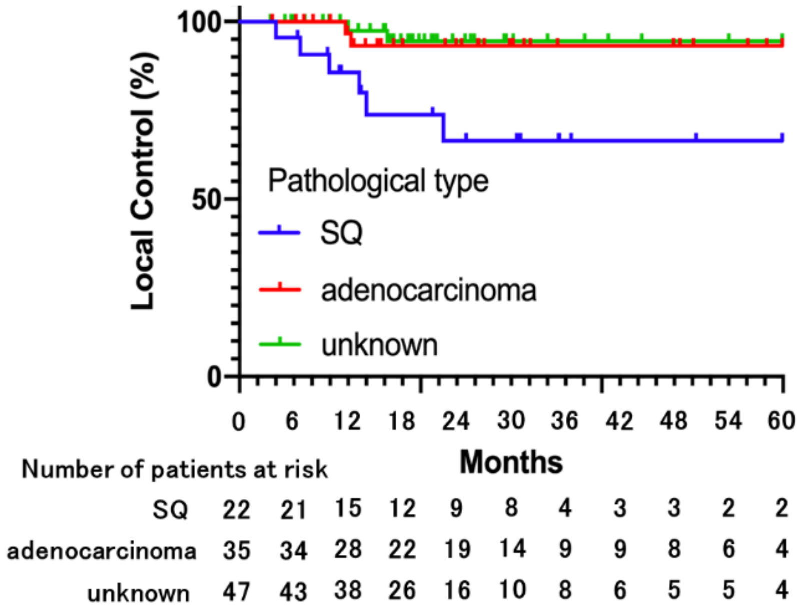

Figure S1: Cumulative rate of local control per pathological diagnosis.
There were significant differences between pathologically proven squamous cell carcinoma (SQ) and pathologically proven adenocarcinoma and pathologically unknown, clinically diagnosed lung cancer (P = 0.022 and 0.009, respectively). However, there were no significant differences between pathologically proven adenocarcinoma and pathologically unknown–clinically diagnosed lung cancer (P = 0.805).
